# A Water-Processed Mesoscale Structure Enables 18.5% Efficient Binary Layer-by-Layer Organic Solar Cells

**DOI:** 10.3390/polym16010091

**Published:** 2023-12-28

**Authors:** Chen Xie, Hui Huang, Zijian Li, Xianghui Zeng, Baoshen Deng, Chengsheng Li, Guangye Zhang, Shunpu Li

**Affiliations:** College of New Materials and New Energies, Shenzhen Technology University, Shenzhen 518118, China; huanghui@sztu.edu.cn (H.H.); 202100304009@stumail.sztu.edu.cn (Z.L.); 2210412044@email.szu.edu.cn (X.Z.); 202100302028@stumail.sztu.edu.cn (B.D.); 202100304012@stumail.sztu.edu.cn (C.L.); zhangguangye@sztu.edu.cn (G.Z.)

**Keywords:** organic solar cells, layer-by-layer processing, eco-friendly water-processing, nanoparticulate mesoscale structure

## Abstract

The two-step layer-by-layer (LBL) deposition of donor and acceptor films enables desired vertical phase separation and high performance in organic solar cells (OSCs), which becomes a promising technology for large-scale printing devices. However, limitations including the use of toxic solvents and unpredictable infiltration between donor and acceptor still hinder the commercial production of LBL OSCs. Herein, we developed a water-based nanoparticle (NP) ink containing donor polymer to construct a mesoscale structure that could be infiltrated with an acceptor solution. Using non-halogen o-xylene for acceptor deposition, the LBL strategy with a mesoscale structure delivered outstanding efficiencies of 18.5% for binary PM6:L8-BObased LBL OSCs. Enhanced charge carrier mobility and restricted trap states were observed in the meso-LBL devices with optimized vertical morphology. It is believed that the findings in this work will bring about more research interest and effort on eco-friendly processing in preparation for the industrial production of OSCs.

## 1. Introduction

Properties including solution printability, semitransparency, and lightweight have piqued the interest of researchers in the realm of organic solar cells (OSCs) [1,2]. In contrast to other new-generation photovoltaics, the eco-friendliness of organic semiconductors enables unique applications of OSCs such as wearable electronics, indoor energy harvesting, building integrated photovoltaics, greenhouses, and so on [3,4,5,6]. Since the introduction of the bulk heterojunction (BHJ) configuration, this architecture has been widely used to fabricate high-performance OSCs [7,8]. Advances in material innovation, interface engineering, and a deeper understanding of morphological physics have enabled the efficiencies of BHJ OSCs to exceed 19% [9,10,11,12]. However, conventional methods for achieving the BHJ architecture involve casting a pre-mixed solution of polymer donor and non-fullerene acceptor (NFA), resulting in a complicated three-phase interplay during film deposition that is dictated by the different crystallization and thermodynamic properties of the materials. This approach often yields an inadequate vertical component distribution, which compromises effective charge transfer and collection [13]. Additionally, the rapid evaporation of the solvent during film deposition can cause kinetically trapped “islands” that are prone to pronounced phase separation caused by the entropic driving force, reducing the stability of the resulting solar cells during device operation [14].

Two-step layer-by-layer (LBL) processing is a representative approach for solving the above concerns. During the deposition of the top layer on the bottom layer, the swelling of the bottom layer induces the interdiffusion between different components and enables the formation of a functional heterojunction framework [15]. This provides an opportunity to overcome the limitations arising from intrinsic thermodynamic properties of materials with insufficient miscibility [16,17]. However, the LBL strategy still faces limitations such as inadequate inter-diffusion during upper layer deposition, which induces unexpected vertical phase separation and phase purity and causes degradation in device performance [14,18,19]. To address these limitations, new controlling techniques must be developed to optimize the vertical morphology for achieving high efficiencies.

The use of mesoscopic materials with controlled pore sizes, shapes, and macrostructures has had a significant impact in various fields, including medicine [20], biology [21], catalysis [21], optics [22], energy storage, and conversion. In addition, it has played a key role in the development of several novel photovoltaic cells, such as dye-sensitized solar cells (DSSCs) [23], quantum dot solar cells (QDSCs) [24], and perovskite solar cells (PSCs) [25,26]. The mesoporous structure is particularly useful in interface engineering for PSCs, as it enlarges the intimate interface for charge extraction and improves the wetting properties for homogenous top-layer coverage. In fact, previous work has demonstrated the use of an eco-friendly processing of organic semiconductor nanoparticles (NPs) to deliver a mesoscale-structured interlayer for PSCs [25]. This approach allows for efficient infiltration of organic porous structures and optimized vertical morphology at the perovskite interface, leading to excellent device performance.

Herein, we report an NP-based mesoscale structure for manipulating the vertical phase separation in LBL-processed OSCs. The nanoparticle (NP) ink with donor was synthesized with an emulsification approach in water and is demonstrated to form a mesostructured NP bottom layer, demonstrating the eco-friendly processing of OSCs. The mesoscale structure allows an in-depth interdiffusion between the bottom and top layers during the solution-processing of the acceptor. Using non-halogen o-xylene for acceptor deposition, the PM6/L8-BO-based Meso-LBL devices yield a PCE of 18.5%, which is one of the best performances among binary LBL OSCs processed with hydrocarbon solvents. The following device physics investigation clarified the charge transport and recombination behaviors in Meso-LBL OSCs. The water-based Meso-LBL strategy addresses the solubility problem of donor polymer and points out a direction for developing NFA materials with high solubility in “green” solvents for LBL processing.

## 2. Materials and Methods

### 2.1. Materials

PM6 and L8-BO were purchased from Solarmer Material Inc (Beijing, China). 1-Dodecyl-3-Methylimidazolium Bromide (DIMMBr) was purchased from Alfa Aesar (Shanghai, China). PEDOT:PSS (CleviosTM P VP AI 4083) was purchased from Heraeus Materials Technology Shanghai, Innochem (Shanghai, China). The other materials and solvents were commercially available and used as received.

### 2.2. NP Synthesis

PM6 5 mg was dissolved in 1mL chloroform and stirred at 50 °C for 3 h. Then, the solution was added to 10 mL 10 mg mL^−1^ DIMMBr aqueous solution and stirred at 40 °C for 1 h. The formed micro-emulsion dispersion was ultrasonicated using a SCIENTZ-IID ultrasonic finger (Scientz Biotechnology Co., Ltd., Ningbo, China) in a water bath. The mini-emulsion system was constantly stirred until chloroform was completely eliminated. The excess surfactant from the particle solution was removed using an Amicon^®^ ultra-15 centrifuge filter (cutoff 30 K) (Sigma Aldrich, St. Louise, MO, USA). The dispersion was placed into the filter and centrifuged at 4000 rpm for 20 min. The retentate was raised to 15 mL with water and then centrifuged again. This process was repeated several times until the surface tension of the filtrate reached 38 ± 2 mN m^−1^. The retentate was filtered with a 0.45 μm filter before the last centrifugation.

### 2.3. Characterization

Particle size and distribution were determined with dynamic light scattering (DLS) using a Malvern Zetasizer Nano ZS90 from Malvern Panalytical (Malvern, UK). UV/Vis absorption spectra were measured with a UV-Vis-NIR spectrometer (Lambda 1050, from Perkin Elmer, Livingston, UK). Scanning electron microscope (SEM) results were obtained with a field emission scanning electron microscope (FESEM) GeminiSEM 300 (Carl Zeiss Microscopy Ltd., Jena, Germany). Atomic Force Microscope (AFM) results were performed by Cypher S from Oxford Instruments Asylum Research, Inc., Oxford, UK in contact mode.

### 2.4. Solar Cells Fabrication and Characterization

Solar cell devices were fabricated in the conventional structure of ITO/PEDOT: PSS/PM6/L8-BO/PNDIT-F3N/Ag. The pre-structured indium tin oxide (ITO) substrates were cleaned with acetone and isopropyl alcohol in an ultrasonic bath for 10 min each. After drying, the substrates were coated with 30 nm of PEDOT:PSS using spin-coating at 5000 rpm and annealed at 180 °C for 15 min. For the LBL devices, a 10 mg mL^−1^ PM6 o-xylene solution was first deposited at 1800 rpm on PEDOT:PSS followed by deposing 8 mg mL^−1^ L8-BO solution at 2000 rpm. Before active layer-processing in Meso-LBL devices, pure toluene was spin-rinsed on the PEDOT:PSS layer. Then, an aqueous donor NP ink of 20 mg mL^−1^ was spin-coated onto a heated PEDOT:PSS substrate (80 °C) at 1250 rpm for 1 min in air. To remove the remaining surfactant, the substrate was spin-rinsed with pure ethanol at 2000 rpm 3 times. Then, the substrate was immediately placed into a N_2_-filled glovebox and stored overnight. After thermal annealing, the acceptor solution of 8 mg mL^−1^ in o-xylene was spin-coated at 2000 rpm. The substrate was then annealed at 100 °C for 5 min.

After active layer deposition, a 0.5 mg mL^−1^ methanol solution of PNDIT-F3N was deposited at 3000 rpm for 30 s in a nitrogen atmosphere, followed by thermal evaporation of 100 nm of silver through a shadow mask with a 6 mm^2^ active area opening under a vacuum of approximately 1 × 10^−6^ mbar. The current–voltage characteristics of the solar cells were measured under AM 1.5 G irradiation on a Newport solar simulator (Taiwan, China). The light source was calibrated using a silicon reference cell. All the cells were tested under an inert atmosphere. EQEs were measured using an Enlitech QE−S EQE system (Taiwan, China) that was equipped with a standard Si diode. Monochromatic light was generated from a Newport 300 W lamp source. The photo-stability of the solar cells was performed under continuous one-sun illumination in a home-built chamber filled with N_2_. Single carrier devices were fabricated, and the dark J–V characteristics were measured and analyzed in the SCL regime following the references.

### 2.5. Space-Charge-Limited Current (SCLC)

The architecture of the hole-only devices was glass/ITO/PEDOT:PSS (30 nm)/active layer/MoO_x_ (10 nm)/Ag (100 nm). The architecture of the electron-only devices was glass/ITO/ZnO (30 nm)/active layer/Ca (5 nm)/Ag (100 nm). The reported mobility data are average values over the 18 devices of each sample for a range of thicknesses. The SCLC curves can be fit to the Mott–Gurney relation for SCLC [27]:

(1) $$J_{SCL}=\frac{9}{8}\varepsilon_{0}\varepsilon_{r}\mu \frac{V_{in}^{2}}{L^{3}}exp\left(\frac{0.89\beta }{L^{0.5}}V_{in}^{0.5}\right)$$

where *J_SCL_* is the current density, *ε*_0_*ε_r_* is the dielectric permittivity, *µ* is the carrier mobility, *L* is the film device, and *β* is the field activation factor [28].

### 2.6. Film-Depth-Dependent Light Absorption Spectra

Film-depth-dependent light absorption spectra were acquired with an in situ spectrometer (PU100, Shaanxi Puguang Weishi Co., Ltd.) (Shaanxi, China) equipped with a soft plasma-ion source. The power supply for generating the soft ionic source was 100 W 39 with an input oxygen pressure of ~10 Pa. The film surface was incrementally etched with the soft ion source, without damage to the materials underneath the surface, which was in situ monitored with a spectrometer. From the evolution of the spectra and the Beer–Lambert’s Law, film-depth-dependent absorption spectra were extracted. The exciton generation contour is numerically simulated upon inputting sub-layer absorption spectra into a modified optical transfer-matrix approach. The detailed experimental and numerical methods are available elsewhere [29,30].

The exciton formation of FLAS measurement is expressed by the absorption coefficient. The absorption coefficient can be obtained according to:

(2) α=4πk/λ

(3) $$I\approx I_{0}e^{-\alpha (\lambda )d}$$

(4) $$A(\lambda )=\mathrm{lg}(I_{0}/I)=-\mathrm{lg}(e^{-4\pi kd/\lambda })$$

The real part (n) of the complex index of refraction is set to be 2 for clarity, while the imaginary part (*k*) of complex refraction is from the absorption coefficient (*α*) and absorbance (*A*). *λ* and d are the wavelength and film thickness. k is related to the film depth. The detailed model is illustrated elsewhere [31].

## 3. Results and Discussion

### 3.1. Meso-LBL Processing

**Figure 1:**
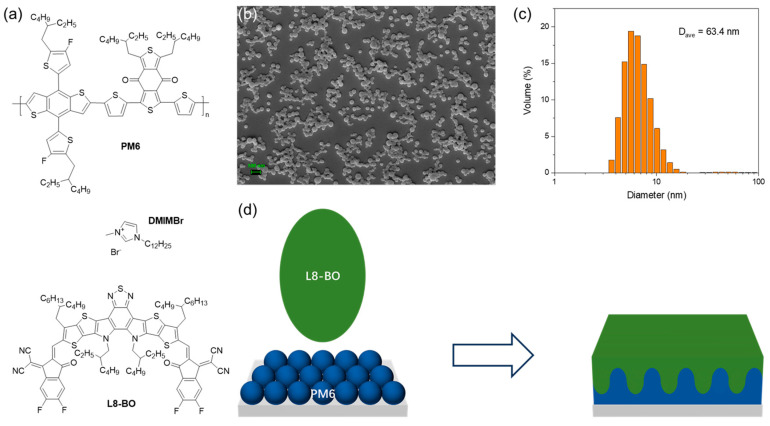
(**a**) Chemical structure of donor PM6, NFA L8-BO, and surfactant DIMMBr. (**b**) SEM of dried PM6 NPs. (**c**) Size distribution of PM6 NPs detected with DLS. (**d**) Schematic diagram of mesostructured-nanoparticle-based layer-by-layer (LBL) polymer:NFA film processed with water and o-xylene for processing the acceptor layer.

The mesostructured bottom layer was constructed by deposition of water-borne NP inks using an emulsification approach. This colloid technique, which was initiated by Landfester et al., exhibits universality in the production of organic NPs for diverse organic electronics such as organic field effect transistors (OFETs), organic photodiodes (OPDs), and organic light-emitting diodes (OLEDs) [32,33]. Thus, we first prove its applicability to formulate water inks for state-of-the-art OSC donors such as PM6 combined with an NFA L8-BO (Figure 1a) [34]. We obtained an oil-in-water emulsion with PM6 droplets after blending the organic solution and water under a vigorous shear force (see Section 2) [28]. An ionized surfactant 1-Dodecyl-3-Methylimidazolium Bromide (DIMMBr) was used to suppress flocculation and Ostwald ripening, leading to a homogenously dispersed NP system with an average sphere diameter of 63 nm (Figure 1b,c).

### 3.2. Meso-LBL Film Characterization

**Figure 2:**
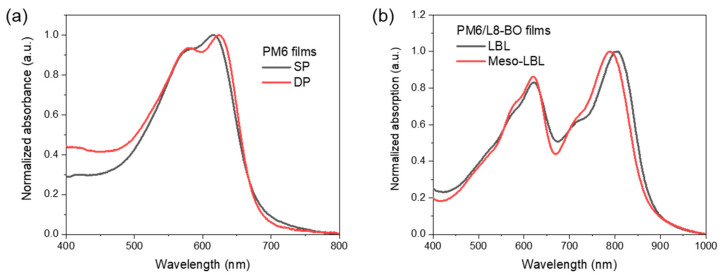
UV−vis absorption spectra of XY and water-processed (**a**) PM6 films and (**b**) PM6/L8-BO films.

The meso-LBL strategy includes firstly processing aqueous donor ink followed by the solution-deposition of the acceptor on top (Figure 1d). The UV-vis absorption spectra of solution-processed (SP) and NP dispersion-processed (DP) PM6 are shown in Figure 2a. The strong UV-vis absorption peak around 620 nm of the dispersion-processed film is the signal of intermolecular π−π stacking, which is expected to facilitate charge transport in the PM6 regions of NP-processed OSCs [35]. The processing of meso-LBL film was finished with the deposition of the semi-orthogonal solvent-carried acceptor solution. As we discussed above, the solvent for top-layer deposition is required to partially dissolve the bottom layer and lead to interpenetrated morphology with graded vertical phase separation. To achieve this, we considered the low-toxic o-xylene (XY) solvent, which proved feasible for PM6-based LBL-processing [8]. After the deposition of L8-BO, the meso-LBL film exhibits a blue shift in the absorption peak of L8-BO around 800 nm, indicating a deep interdiffusion between the donor and the acceptor during the deposition of L8-BO upon mesostructured PM6 (Figure 2b).

**Figure 3:**
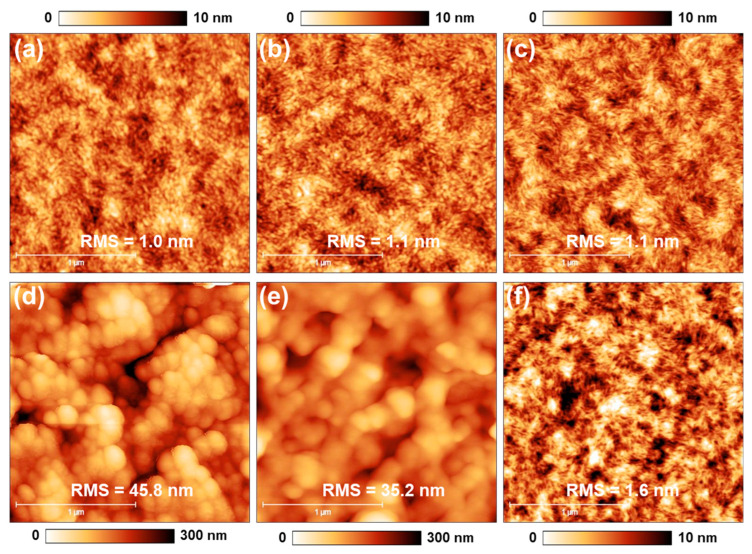
AFM of (**a**,**d**) PM6 films, (**b**,**e**) PM6 films after annealing 100 °C for 5 min, and (**c**,**f**) PM6/L8-BO films with (**a**–**c**) solution-processed (SP) and (**d**–**f**) dispersion-processed (DP) PM6, respectively.

The morphology of NP and LBL films was investigated with AFM measurements. As shown in Figure 3a, SP PM6 exhibits a homogenous surface. However, a mesoporous film with discrete spheres and cavities was formulated after spin-coating PM6 NPs (Figure 3d). Additionally, mild thermal treatment (100 °C, 5 min) gradually sintered the particles and blurs mesopores, which is indicated by the gentled surface roughness (Figure 3e). This morphology evolution can be ascribed to the kinetically accelerated movement of PM6 chains under its glass transition temperature (T_g_) around 120 °C [36,37]. Therefore, it is indicated that the mesoporosity of the meso-PM6 layer can be fine-tuned with a mild thermal treatment in the range of T_g_.

After the deposition of solution-processed L8-BO, both LBL and meso-LBL films showed homogenous surfaces, and the porous structure of NP PM6 disappeared. The roughness of meso-LBL is higher than that of conventional LBL film, suggesting an intimate interface contact with the interlayer.

**Figure 4:**
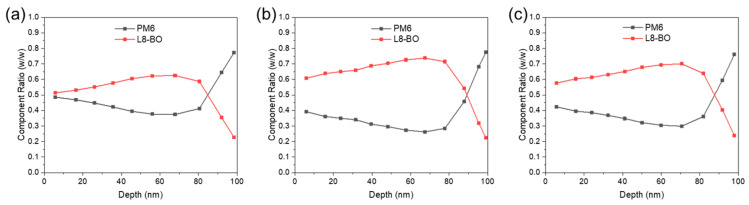
Composition ratio across the vertical direction of the PM6:L8−BO film in (**a**) blend coating, (**b**) LBL, and (**c**) Meso−LBL solar cells.

Vertical phase separation of the donor and acceptor materials in OSCs is crucial for the efficient dissociation of excitons and charge transport. To study the vertical phase morphology of the active layer, we used film-depth-dependent light absorption spectroscopy (FLAS), which was measured in situ by incrementally etching the sample with soft plasma, as previously reported [29,30]. As shown in Figure S1a, the absorption peak of L8-BO around 800 nm in the blend coating film, which is processed by solution with a blended donor and acceptor, varies with film depth, indicating inefficient electron transport caused by low-energy-level regions [38]. As shown in Figure 4, all the active layers exhibit a graded component distribution from the top to the bottom. The blend coating film exhibits the most intermixed vertical morphology because the mass ratios of the donor and acceptor are close to each other (Figure 4a). A more pronounced vertical phase separation of the LBL film in comparison with that of the meso-LBL film indicates an insufficient interpenetration between the PM6 and L8-BO phases (Figure 4b,c). This result suggests that the mesoporous structure facilitates the downshifting movement of L8-BO molecules into the PM6 regions and delivers an active layer with relieved phase separation.

### 3.3. Solar Cell Characterization

**Figure 5:**
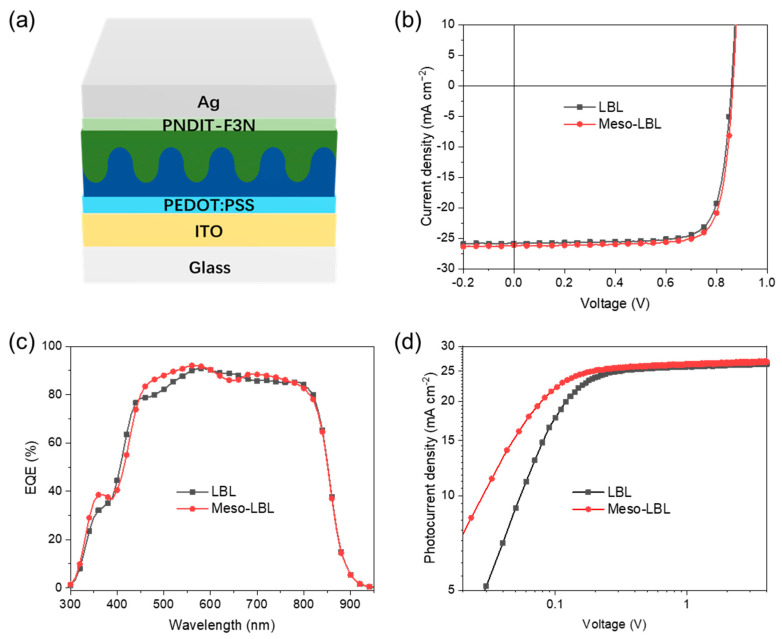
(**a**) Device architecture of the PM6/L8-BO meso-LBL solar cell, (**b**) J−V characteristics, (**c**) EQE, and (**d**) photocurrent of PM6/L8-BO LBL solar cells.

The LBL OSCs based on PM6/L8-BO with a traditional architecture were fabricated along with PEDOT:PSS and PNDIT-F3N as interlayers (Figure 5a). The current density–voltage (J–V) characteristics of meso-LBL devices and corresponding parameters are depicted in Figure 5b and Table 1. The mn-LBL processed OSCs produced a best PCE of 18.45%, with an open-circuit voltage (V_OC_) of 0.88 V, a short-circuit current (J_SC_) of 26.17 Ma cm^−2^, and a fill factor (FF) of 79.76%, which is much higher than the value of 17.75% in conventional LBL devices. As shown in Table 1, the PCE enhancement in mn-LBL devices is mainly caused by the high J_SC_ and FF values in comparison with a J_SC_ value of 25.94 Ma cm^−2^ and an FF of 78.28% for conventional LBL solar cells. The V_OC_ of LBL devices is only 0.1 V less than that of meso-LBL devices. In comparison, the blend coating device processed with the PM6:L8-BO blended solution was also fabricated and exhibits a champion PCE of 17.77%, with V_OC_, J_SC_, and FF values of 0.87 V, 25.71 mA cm^−2^, and 79.33%, respectively. The J-V curve of blend coating PM6:L8-BO solar cells is shown in Figure S2. It is indicated that the performance of blend coating devices is comparable to that of conventional LBL solar cells. Additionally, the dark J-V curves of meso-LBL devices show slightly less current leakage in comparison with conventional LBL ones (Figure S3). The fitted series resistance (R_s_) and shunt resistance (R_sh_) for conventional LBL solar cells are 12.2 Ω cm^2^ and 6.7 Kω cm^2^, respectively. The meso-LBL solar cells delivered an R_s_ of 10.8 Ω cm^2^ and an R_sh_ of 10.4 Kω cm^2^, respectively. Thus, the decreased R_s_ and increased R_sh_ due to the introduction of mesoscale structure in LBL solar cells play pivotal roles in increasing the FF and PCE. The J_SC_s integrated from the external quantum efficiency (EQE) spectra demonstrate less than 2% mismatch to those obtained from the J-V curves (Figure 5c). The high EQE around 500–600 nm suggests efficient photovoltaic conversion of PM6 in meso-LBL solar cells, which is in line with the enhanced Jsc value. As shown in Figure 5d, the increase in photocurrent (J_ph_) under effective reverse bias (V_in_) is more significant in LBL devices than in meso-LBL ones. The photocurrent (J_ph_ @ −5 V) saturates at 26.44 and 26.89 Ma cm^−2^ for LBL and meso-LBL devices, respectively. The high photocurrent and its electrical field dependence are beneficial for high J_SC_ and FF values in meso-LBL devices. Stronger electrical field dependence in meso-LBL solar cells could be due to more efficient separation of bound e–h pairs (geminate recombination) or reduced recombination of free charges (non-geminate recombination) [28].

### 3.4. Device Physics Analysis

**Figure 6:**
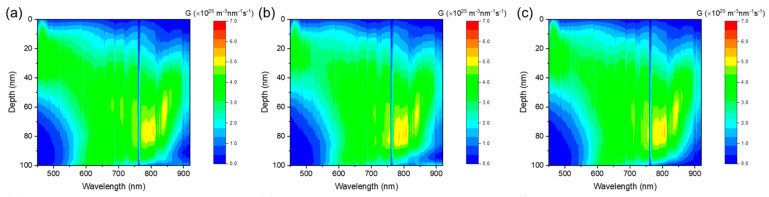
Exciton generation contours of the active layer in (**a**) blend coating, (**b**) LBL, and (**c**) Meso-LBL solar cells.

The FLAS measurement was further applied to simulate the exciton generation behaviors in the LBL active layers. As shown in Figure 6, the majority of excitons were generated by L8-BO around 20 nm to the bottom PEDOT:PSS layer, indicating that the electrons dissociated from acceptor-generated excitons prefer a high value of mobility to transport across the active layer and reach the cathode. Hence, electron mobility determines the device performance in all three types of PM6:L8-BO devices.

**Figure 7:**
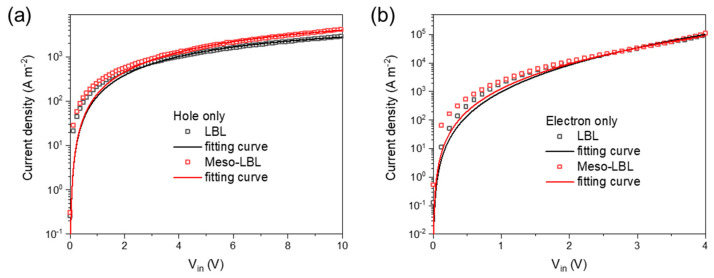
Dark J-V characteristics of PM6/L8-BO based (**a**) hole-only and (**b**) electron-only devices. Hole-only device structure: glass/ITO/PEDOT:PSS/NPs/MoOx/Ag; electron-only device structure: glass/ITO/ZnO/NPs/Ca/Ag.

The charge-carrier mobilities of meso-LBL solar cells were tested with the SCLC model with the fabrication of hole-only and electron-only devices (see Section 2). As depicted in Figure 7a, the conventional LBL device exhibits a relatively low hole mobility of 8.75 × 10^−5^ cm^2^ V^−1^ s^−1^ in comparison with that of the meso-LBL device (9.10 × 10^−5^ cm^2^ V^−1^ s^−1^). In addition, the values of electron mobility (μ_e_) exhibit a large difference between these two types of devices, which exhibit a μ_e_ of 5.05 × 10^−5^ cm^2^ V^−1^ s^−1^ and 9.49 × 10^−5^ cm^2^ V^−1^ s^−1^ for conventional LBL and meso-LBL solar cells, respectively. These results are in agreement with exciton-generation behaviors that electron mobility determines the device’s performance. The high μ_e_ of meso-LBL devices with a balanced μ_h_ and μ_e_ ratio (Table 1) is beneficial to charge extraction and results in an improvement in the Jsc and FF values.

**Figure 8:**
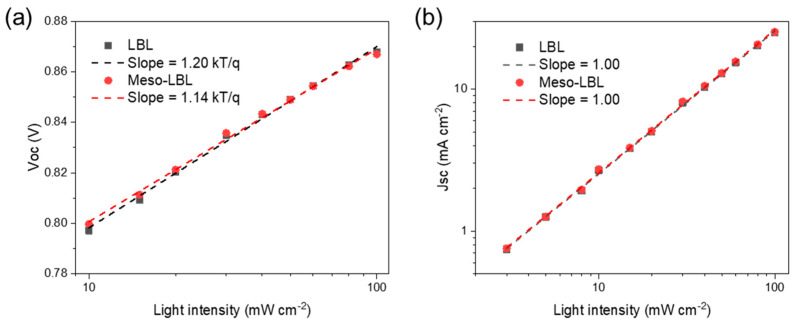
(**a**) V_OC_ (**b**) J_SC_ and as a function of light intensity for PM6/L8-BO LBL solar cells.

The charge trapping behaviors, which usually cause severe non-radiative recombination and affect the carrier diffusion and transport in OSCs, were examined using the dependence of V_OC_ on the light intensity of meso-LBL devices (Figure 8). The conventional LBL and meso-LBL devices exhibit slope values of 1.20 and 1.14 Kt/q, respectively, where k is the Boltzmann constant, T is Kelvin temperature, and q is the elementary charge [39]. According to a previous report, when the slope is close to 2, the decay of V_OC_ is a second-order dependence upon charge density [40]. However, slopes for both devices are close to 1, indicating an approximately first-order recombination behavior. The conventional LBL devices suffer more from the first-ordered trap-assisted recombination under the open-circuit condition. However, J_SC_ dependence upon light intensity for both types of devices exhibits fitted slopes of 1.00, indicating that the J_SC_ reduction in the conventional LBL device is not mainly caused by bimolecular recombination. The light intensity dependence result reveals that the V_OC_ loss in conventional LBL solar cells is mainly caused by first-ordered trap-assisted recombination.

**Figure 9:**
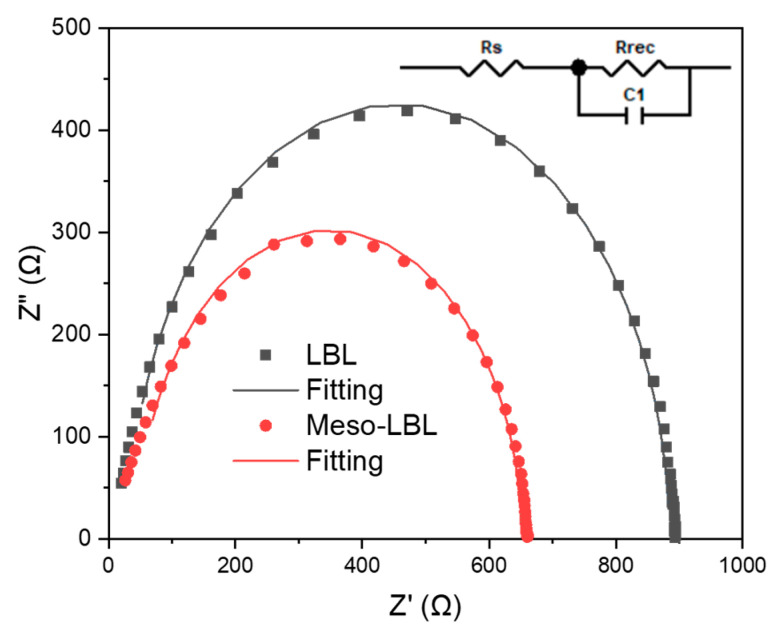
Nyquist plot of the PM6/L8-BO LBL solar cells.

To gain a deep understanding of the charge transport and recombination processes in the LBL active layers, electrochemical impedance spectroscopy (EIS) measurements were carried out in a frequency range from 100 Hz to 1 MHz in dark and at open circuit bias conditions [41]. Figure 9 shows the corresponding Nyquist plots of the optimized LBL solar cells measured at V = V_OC_. The solid lines represent the fitting results derived from the equivalent circuit model, as shown in the insert of Figure 9. According to the equivalent circuit model, the fitting parameters are listed in Table 1. The Rs values are reduced from 46.0 Ω for conventional LBL device to 31.9 Ω for the meso-LBL solar cells, while the R_rec_ values in LBL device are significantly increased from 604.7 Ω to 856.1 Ω after using meso-LBL processing. The reduced Rs and increased R_rec_ indicate the facilitated charge transport and suppressed charge recombination in the meso-LBL solar cells, which can well explain the increased FF values.

## 4. Conclusions

In summary, we successfully introduced a water-borne NP ink of donor to construct a mesoporous interface for the LBL processing of OSCs. This elegant meso-LBL strategy promotes interdiffusion between the donor and acceptor, resulting in an active layer with optimized vertical morphology. Eco-friendly solar cells based on water-processed meso-PM6 and hydrocarbon XY-processed L8-BO delivered a champion PCE of 18.5%. UV−vis absorption spectra and FLAS measurements demonstrate enhanced interdiffusion between the donor and acceptor in meso-LBL OSCs. V_OC_ dependence upon light intensity indicates that trap states result in severe Voc loss in conventional LBL devices. SCLC shows inefficient electron transport and pronounced charge recombination from solar cells under conventional LBL processing. This is the first time that OSCs were fabricated with the water-processing of a donor. This meso-LBL strategy addresses the solubility problem of donor polymers and points out a direction for developing NFA materials with high solubility in a “green” solvent for LBL processing. The application of this strategy for OSCs is promising to overcome the processing limitation of LBL technology, such as insufficient interdiffusion between the donor and acceptor and unpredictable vertical phase morphology. We believe that the meso-LBL approach, combined with a reasonable design of NFA molecules, can enable the eco-friendly production of large-scale OSCs using printing techniques.

## Figures and Tables

**Table 1:** Summary of the photovoltaic parameters, saturate photocurrent, and charge carrier mobility values of the PM6:L8-BO-based layer-by-layer solar cells.

Processing Method	V_OC_ (V)	J_SC_ (mA cm^−2^)	J_SC_ (mA cm^−2^) ^1^	FF (%)	PCE (%)	J_sat_ (mA cm^−2^)	μ_h_ (cm^2^ V^−1^ s^−1^)	μ_e_ (cm^2^ V^−1^ s^−1^)	μ_h_/μ_e_	R_S_ (Ω)	R_rec_ (Ω)
LBL	0.87	25.94	25.56	78.28	17.75	26.44	8.75 × 10^−5^	5.05 × 10^−5^	1.73	46.0	604.7
Meso−LBL	0.88	26.17	25.80	79.76	18.45	26.89	9.10 × 10^−5^	9.49 × 10^−5^	0.96	31.9	851.6

^1^ J_SC_ values calculated from the EQEs of devices with the best PCEs.

## Data Availability

Data are contained within the article.

## Supplementary Materials

The following supporting information can be downloaded at: https://www.mdpi.com/article/10.3390/polym16010091/s1.

## References

[1] Spyropoulos G.D., Kubis P., Li N., Baran D., Lucera L., Salvador M., Ameri T., Voigt M.M., Krebs F.C., Brabec C.J. (2014). Flexible organic tandem solar modules with 6% efficiency: Combining roll-to-roll compatible processing with high geometric fill factors. Energy Environ. Sci..

[2] Kaltenbrunner M., White M.S., Głowacki E.D., Sekitani T., Someya T., Sariciftci N.S., Bauer S. (2012). Ultrathin and lightweight organic solar cells with high flexibility. Nat. Commun..

[3] Krebs F.C., Nielsen T.D., Fyenbo J., Wadstrøm M., Pedersen M.S. (2010). Manufacture, integration and demonstration of polymer solar cells in a lamp for the “lighting Africa” initiative. Energy Environ. Sci..

[4] Ryu H.S., Park S.Y., Lee T.H., Kim J.Y., Woo H.Y. (2020). Recent progress in indoor organic photovoltaics. Nanoscale.

[5] Meitzner R., Schubert U.S., Hoppe H. (2021). Agrivoltaics—The Perfect Fit for the Future of Organic Photovoltaics. Adv. Energy Mater..

[6] Lee B., Lahann L., Li Y., Forrest S.R. (2020). Cost estimates of production scale semitransparent organic photovoltaic modules for building integrated photovoltaics. Sustain. Energy Fuels.

[7] Jiang K., Zhang J., Peng Z., Lin F., Wu S., Li Z., Chen Y., Yan H., Ade H., Zhu Z. (2021). Pseudo-bilayer architecture enables high-performance organic solar cells with enhanced exciton diffusion length. Nat. Commun..

[8] Zhang Y., Liu K., Huang J., Xia X., Cao J., Zhao G., Fong P.W.K., Zhu Y., Yan F., Yang Y. (2021). Graded bulk-heterojunction enables 17% binary organic solar cells via nonhalogenated open air coating. Nat. Commun..

[9] Zhu L., Zhang M., Xu J., Li C., Yan J., Zhou G., Zhong W., Hao T., Song J., Xue X. (2022). Single-junction organic solar cells with over 19% efficiency enabled by a refined double-fibril network morphology. Nat. Mater..

[10] Wei Y., Chen Z., Lu G., Yu N., Li C., Gao J., Gu X., Hao X., Lu G., Tang Z. (2022). Binary Organic Solar Cells Breaking 19% via Manipulating the Vertical Component Distribution. Adv. Mater..

[11] He C., Pan Y., Ouyang Y., Shen Q., Gao Y., Yan K., Fang J., Chen Y., Ma C.Q., Min J. (2022). Manipulating the D:A interfacial energetics and intermolecular packing for 19.2% efficiency organic photovoltaics. Energy Environ. Sci..

[12] Sun R., Wu Y., Yang X., Gao Y., Chen Z., Li K., Qiao J., Wang T., Guo J., Liu C. (2022). Single-Junction Organic Solar Cells with 19.17% Efficiency Enabled by Introducing One Asymmetric Guest Acceptor. Adv. Mater..

[13] Yu R., Wei X., Wu G., Tan Z. (2022). Layer-by-layered organic solar cells: Morphology optimizing strategies and processing techniques. Aggregate.

[14] Jee M.H., Ryu H.S., Lee D., Lee W., Woo H.Y. (2022). Recent Advances in Nonfullerene Acceptor-Based Layer-by-Layer Organic Solar Cells Using a Solution Process. Adv. Sci..

[15] Fu H., Peng Z., Fan Q., Lin F.R., Qi F., Ran Y., Wu Z., Fan B., Jiang K., Woo H.Y. (2022). A Top-Down Strategy to Engineer ActiveLayer Morphology for Highly Efficient and Stable All-Polymer Solar Cells. Adv. Mater..

[16] Ghasemi M., Ye L., Zhang Q., Yan L., Kim J.H., Awartani O., You W., Gadisa A., Ade H. (2017). Panchromatic Sequentially Cast Ternary Polymer Solar Cells. Adv. Mater..

[17] Li M., Wang Q., Liu J., Geng Y., Ye L. (2021). Sequential deposition enables high-performance nonfullerene organic solar cells. Mater. Chem. Front..

[18] Zhan L., Li S., Xia X., Li Y., Lu X., Zuo L., Shi M., Chen H. (2021). Layer-by-Layer Processed Ternary Organic Photovoltaics with Efficiency over 18%. Adv. Mater..

[19] Cai Y., Li Q., Lu G., Ryu H.S., Li Y., Jin H., Chen Z., Tang Z., Lu G., Hao X. (2022). Vertically optimized phase separation with improved exciton diffusion enables efficient organic solar cells with thick active layers. Nat. Commun..

[20] Mamaeva V., Sahlgren C., Lindén M. (2013). Mesoporous silica nanoparticles in medicine-Recent advances. Adv. Drug Deliv. Rev..

[21] Daniel M.C., Astruc D. (2004). Gold Nanoparticles: Assembly, Supramolecular Chemistry, Quantum-Size-Related Properties, and Applications Toward Biology, Catalysis, and Nanotechnology. Chem. Rev..

[22] Luo T., Huang P., Gao G., Shen G., Fu S., Cui D., Zhou C., Ren Q. (2011). Mesoporous silica-coated gold nanorods with embedded indocyanine green for dual mode X-ray CT and NIR fluorescence imaging. Opt. Express.

[23] Kokkonen M., Talebi P., Zhou J., Asgari S., Soomro S.A., Elsehrawy F., Halme J., Ahmad S., Hagfeldt A., Hashmi S.G. (2021). Advanced research trends in dye-sensitized solar cells. J. Mater. Chem. A.

[24] Zhao Q., Han R., Marshall A.R., Wang S., Wieliczka B.M., Ni J., Zhang J., Yuan J., Luther J.M., Hazarika A. (2022). Colloidal Quantum Dot Solar Cells: Progressive Deposition Techniques and Future Prospects on Large-Area Fabrication. Adv. Mater..

[25] Hou Y., Xie C., Radmilovic V.V., Puscher B., Wu M., Heumüller T., Karl A., Li N., Tang X., Meng W. (2019). Assembling Mesoscale-Structured Organic Interfaces in Perovskite Photovoltaics. Adv. Mater..

[26] Foo S., Thambidurai M., Senthil Kumar P., Yuvakkumar R., Huang Y., Dang C. (2022). Recent review on electron transport layers in perovskite solar cells. Int. J. Energy Res..

[27] Blom P.W.M., De Jong M.J.M., Vleggaar J.J.M. (1996). Electron and hole transport in poly(p-phenylene vinylene) devices. Appl. Phys. Lett..

[28] Xie C., Classen A., Späth A., Tang X., Min J., Meyer M., Zhang C., Li N., Osvet A., Fink R.H. (2018). Overcoming Microstructural Limitations in Water Processed Organic Solar Cells by Engineering Customized Nanoparticulate Inks. Adv. Energy Mater..

[29] Zhang Y., Deng D., Wang Z., Wang Y., Zhang J., Fang J., Yang Y., Lu G., Ma W., Wei Z. (2017). Enhancing the Photovoltaic Performance via Vertical Phase Distribution Optimization in Small Molecule:PC71BM Blends. Adv. Energy Mater..

[30] Bu L., Gao S., Wang W., Zhou L., Feng S., Chen X., Yu D., Li S., Lu G. (2016). Film-Depth-Dependent Light Absorption and Charge Transport for Polymer Electronics: A Case Study on Semiconductor/Insulator Blends by Plasma Etching. Adv. Electron. Mater..

[31] Feng X., Wang Y., Xiao T., Shen Z., Ren Y., Lu G., Bu L. (2020). In situ Measuring Film-Depth-Dependent Light Absorption Spectra for Organic Photovoltaics. Front. Chem..

[32] Landfester K. (2001). The generation of nanoparticles in miniemulsions. Adv. Mater..

[33] Cho J., Yoon S., Min Sim K., Jin Jeong Y., Eon Park C., Kwon S.K., Kim Y.H., Chung D.S. (2017). Universal selection rule for surfactants used in miniemulsion processes for eco-friendly and high performance polymer semiconductors. Energy Environ. Sci..

[34] Cai Y., Li Y., Wang R., Wu H., Chen Z., Zhang J., Ma Z., Hao X., Zhao Y., Zhang C. (2021). A Well-Mixed Phase Formed by Two Compatible Non-Fullerene Acceptors Enables Ternary Organic Solar Cells with Efficiency over 18.6%. Adv. Mater..

[35] Zhang Y., Ma R., Wang Y., Wang Y., Liu T., Xiao Y., Cho Y., Luo Z., Lu X., Zhao L. (2021). Highly crystalline acceptor materials based on benzodithiophene with different amount of fluorine substitution on alkoxyphenyl conjugated side chains for organic photovoltaics. Mater. Rep. Energy.

[36] Ulum S., Holmes N., Barr M., Kilcoyne D., Bin B., Zhou X., Belcher W., Dastoor P. (2013). The role of miscibility in polymer: Fullerene nanoparticulate organic photovoltaic devices. Nano Energy.

[37] Holmes N.P., Marks M., Kumar P., Kroon R., Barr M.G., Nicolaidis N., Feron K., Pivrikas A., Fahy A., Mendaza A.D. (2016). de Z.; et al. Nano-pathways: Bridging the divide between water-processable nanoparticulate and bulk heterojunction organic photovoltaics. Nano Energy.

[38] Wang Z., Hu Y., Xiao T., Zhu Y., Chen X., Bu L., Zhang Y., Wei Z., Xu B.B., Lu G. (2019). Correlations between Performance of Organic Solar Cells and Film-Depth-Dependent Optical and Electronic Variations. Adv. Opt. Mater..

[39] Koster L.J.A., Mihailetchi V.D., Ramaker R., Blom P.W.M. (2005). Light intensity dependence of open-circuit voltage of polymer:fullerene solar cells. Appl. Phys. Lett..

[40] Shuttle C.G., O’Regan B., Ballantyne A.M., Nelson J., Bradley D.D.C., Durrant J.R. (2008). Bimolecular recombination losses in polythiophene: Fullerene solar cells. Phys. Rev. B.

[41] Garcia Romero D., Di Mario L., Yan F., Ibarra-Barreno C.M., Mutalik S., Protesescu L., Rudolf P., Loi M.A. (2023). Understanding the Surface Chemistry of SnO_2_ Nanoparticles for High Performance and Stable Organic Solar Cells. Adv. Funct. Mater..

