# Supplementary material for: A Water-Processed Mesoscale Structure Enables 18.5% Efficient Binary Layer-by-Layer Organic Solar Cells
Source: Polymers (Basel). 2023 Dec 28;16(1):91. doi: 10.3390/polym16010091 (PMC10780782; doi:10.3390/polym16010091)
Supplement: Supplementary file 1 [file polymers-16-00091-s001.zip › polymers-2789835-supplementary.pdf]

Water-processed mesoscale structure enables 18.5% efficient binary layer-by-layer organic solar cells

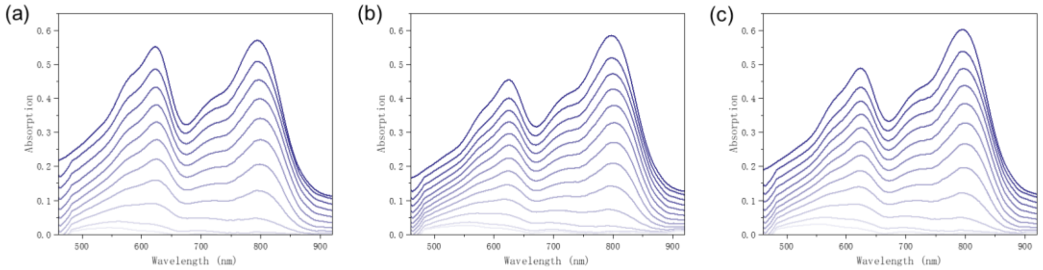

Figure S1. FLAS of (a) BC, (b) LBL and (c) meso-LBL processed PM6:L8-BO films.

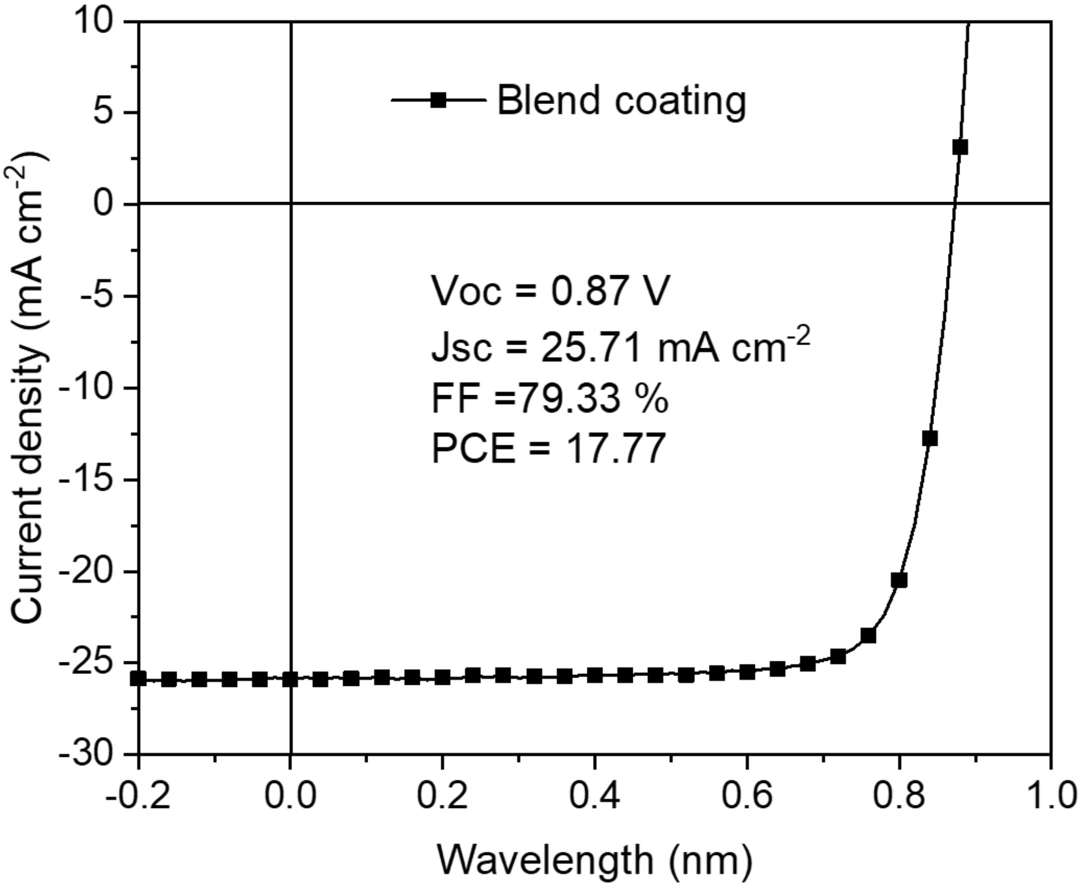

Figure S2. J−V characteristics of blend coating PM6:L8-BO solar cells.

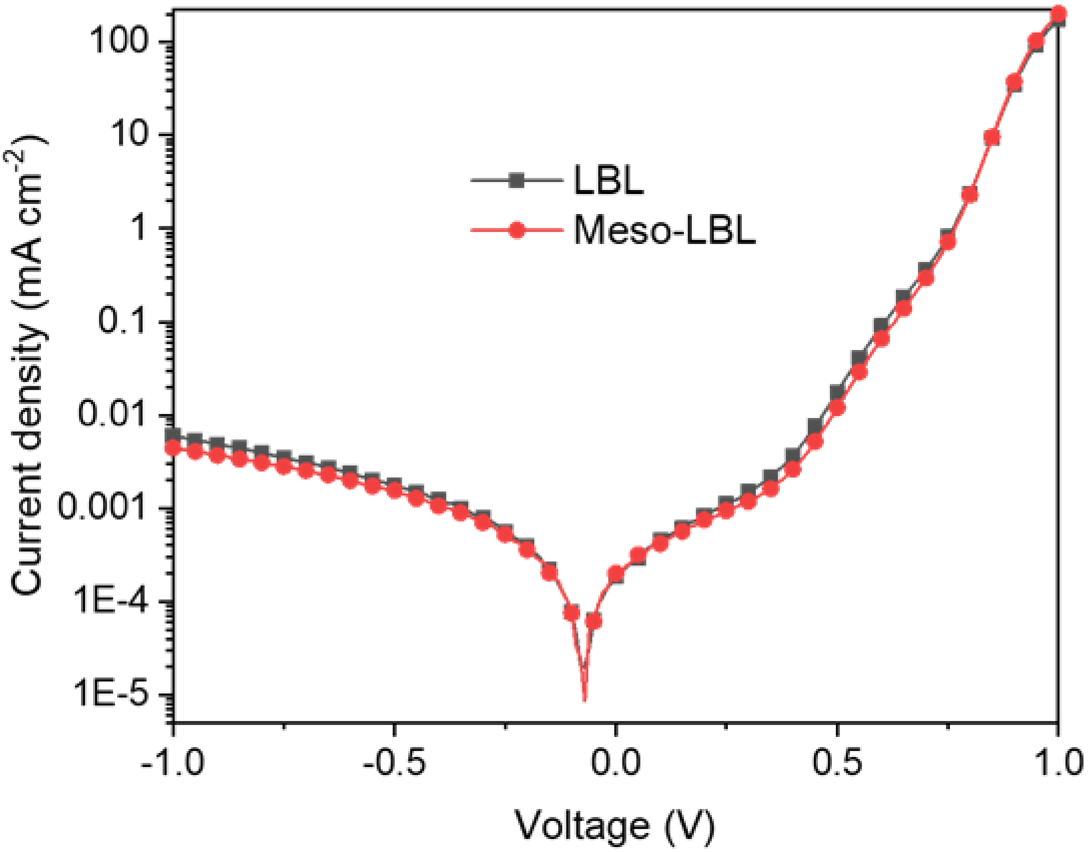

Figure S3. Dark J−V characteristics of PM6/L8-BO conventional LBL and meso-LBL solar cells.
